# Cloning and Functional Assessments of Floral-Expressed *SWEET* Transporter Genes from *Jasminum sambac*

**DOI:** 10.3390/ijms20164001

**Published:** 2019-08-16

**Authors:** Panpan Wang, Peining Wei, Fangfei Niu, Xiaofeng Liu, Hongliang Zhang, Meiling Lyu, Yuan Yuan, Binghua Wu

**Affiliations:** 1 Fujian Provincial Key Laboratory of Plant Functional Biology, College of Horticulture, Fujian A & University, Fuzhou 350002, China; 2 College of Life Sciences, Fujian Agriculture and Forestry University, Fuzhou 350002, China

**Keywords:** SWEET transporter, gene expression, sugar transport, flowers, *Jasminum sambac*

## Abstract

Sugar transporters of the *SWEET* family mediate cross membrane movement of mono- and disaccharides and play vital roles in diverse physiological and pathophysiological processes, including sink–source relationship, pathogen responses, reproductive growth, and development. However, it remains to be determined how these transporters function in non-module plants of agricultural significance, given the evolutionarily diverse traits. In this study, we combined transcriptome analysis, rapid amplification of cDNA ends-cloning (RACE-cloning), expression profiling, and heterologous functional assay to identify *SWEET* genes that may have potential roles during flower opening and sexual reproduction in *Jasminum sambac* . During the anthesis, the floral organs of *J. sambac* express seven *SWEET* homologous genes from all four clades of the family. *JsSWEET9* and *2* are significantly upregulated when flowers are fully opened, up to 6- and 3-fold compared to unopened buds, respectively. The other transporters, *JsSWEET1*, *5*, *10*, and *17* are also accumulated slightly at stage associated with fragrance release, whereas only the vacuole transporter *JsSWEET16* showed small decrease in transcript level after anthesis. The *JsSWEET5*, a clade II member, is capable to complement yeast cell uptake on most tested sugar substrates with a preference for hexoses, while the clade I transporter *JsSWEET1* mediates merely galactose import when expressed in yeast. Our results provide first evidence for further investigation on sugar transport and allocation during flowering and reproductive processes in *J. sambac*.

## 1. Introduction

Sugar transport in higher plants involves at least three major groups of proteins [1]. Members of the monosaccharide/polyols transporters-like (MST) superfamily constitute seven divers subfamilies and transport a wide range of substrates in addition to glucose [2,3,4]. For example, the subfamily of *S*ugar Transport *P*roteins (STP) in Arabidopsis contains fifteen genes, six out of which have been shown to act redundantly in mediating glucose uptake by pollen tubes [5,6]. More importantly, members of the STPs—AtSTP1 and AtSTP13—have been implicated in antibacterial defense by sequestration of apoplasmic sugars to limit pathogens’ nutrition [7,8]. Another major class of transporters belongs to the sucrose transporter/carriers (SUT/SUC) family, which are the first sucrose/H^+^ symporters characterized as being important for phloem loading and unloading processes [9]. This family has nine genes in Arabidopsis, but typically consists of five members in haploid of most monocotyledon species as well as in ancient vascular plants such as *Selaginella*, and one single gene in moss [2]. Homologs of the SUT/SUC family can be classified phylogenetically as three types or five clades [10,11], with the existence of evolutionary isolated subclade members. Many SUTs have demonstrated sucrose and/or maltose transport activities at plasma membrane or tonoplast [11]. Being major players for photosynthetic sucrose translocation and partitioning between source–sink organs, most of the SUT transporters are expressed mainly in phloem tissues of both source (leaves) and sink (roots, flowers, and other storage or reproductive structures) organs, especially in the sieve element-companion cell complex [10,12]. Both MST and SUT/SUC families are closely related to the major facilitator superfamily (MFS).

The third group of sugar transporters identified are the SWEET proteins that mediated bidirectional cross-membrane movement of mono- and disaccharides by an alternating access mechanism [13,14,15,16]. The eukaryotic SWEET proteins have a conserved trimeric structure with seven transmembrane helices that fold into two symmetric N- and C-terminal bundles (triple-helix bundles, THBs), linked by the fourth helix, whereas their prokaryotic counterparts, called SemiSWEETs that also function as mono- or disaccharides transporters, contain only a single THB enduring a dimeric structure [17,18,19,20]. In general, a typical angiosperm genome contains 15 to 25 *SWEET* genes, e.g., 17 are encoded in Arabidopsis and 21 in rice. *SWEET* gene duplication occurs during evolution in numerous plant species, such as Eucalyptus (47 genes) [14], soybean (52) [21], wheat (108) [22], cabbage (30) [23], cultivated cotton (55) [24], and rubber tree (36) [25], contrary to only one single gene encoded by human and Drosophila genomes [16,26]. The four-clade classified SWEET proteins play very important roles in diverse physiological and pathophysiological processes (recent reviews in [14,27,28]). The involvements of different *SWEET* homologs in source–sink relationship [15,29], pathogen responses [16,30,31], nectar secretion [32], and fruit/seed development [33,34,35] are among the well-studied.

The reproductive organs of higher plants, from anthesis to seed maturation, are temporally and spatially strong sinks for sugar allocation and distribution. The importance of sugar transport for the reproduction process is highlighted by the expression of as many as 31 distinct sugar transporter genes in Arabidopsis floral organs [36], and individual transporter may also have critical function. For example, lack of *AtSWEET8* (also called *RPG1*) leads to male sterility [37]. AtSWEET9 functions essentially as a sucrose efflux transporter in the nectaries, together with sucrose synthase (SUS) and cell wall invertases (cwINV), for nectar secretion of a mixture of sugars. Interestingly, silencing the Petunia homolog of *SWEET9*, *PhNEC1*, resulted in malfunction of anther opening and male sterility [38]. However, most of our knowledge about the transport, distribution, metabolism, and signaling of sugars in the reproduction processes are gained from studies in model plants such as Arabidopsis, whilst other plant species may have evolved other mechanisms.

*Jasminum sambac* is a fragrant ornamental plants widely cultivated in the Middle East and South-East Asia for harvest of flowers. Under nature conditions the plant exhibit a very low fruit and seed set ranging from 0.13% to 0.19% due to low pollen viability and possible premature cessation of pistil development that hinders the breeding efforts for variety improvement [39,40]. Despite the horticultural significance, it has not been fully exploited at the molecular level regarding the sexual reproduction of this plant species. Carbohydrate metabolism and regulation during flower growth are among the important issues to be addressed [36]. In this study, we identified seven floral expressed SWEET transporter genes through transcriptome analysis and RACE-cloning. By monitoring their expression during anthesis and characterizing their uptake activity in a yeast complementation assay, we showed that *JsSWEET2* and *JsSWEET9* were the most expressed genes at the later stage of flowering and the JsSWEET5 was able to transport both mono- and disaccharides at the plasma membrane. Our results provides useful information for further deciphering functions of sugar transporters during flowering in this special plant.

## 2. Results

### 2.1. Identification of Seven Flower-Expressed SWEET Genes Representative of the Four-Clade Family

**Figure 1:**
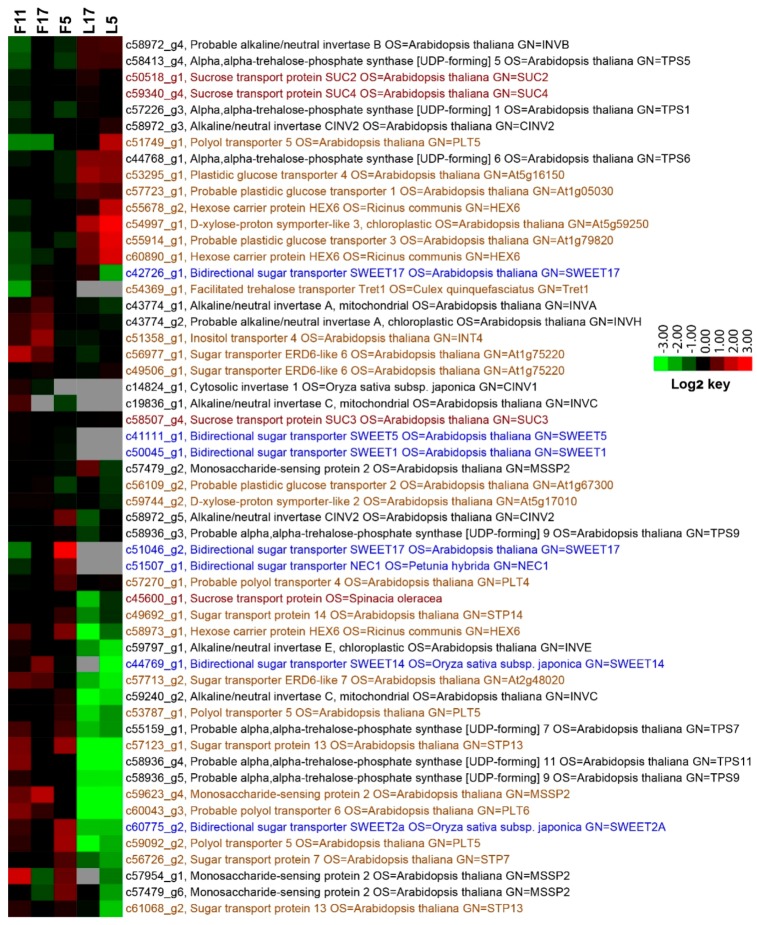
A heat map showing differentially expressed genes for SWEET transporter-like (highlight in blue), sucrose transporter-like (in red) and monosaccharide transporter-like (in orange), invertases-like and trehalose-6-phosphate synthase-like in *J. sambac* flowers and leaves at indicated time of the day. Genes were clustered by uncentered Pearson correlation with average linkage ordering, using Cluster 3.0 (version 1.57). The log_2_-transformed fold change is indicated by the color bar. Samples F11, F17, F5, and L17, L5: flowers at 11:00, 17:00, 05:00, and leaves at 17:00, 05:00.

As a fragrant and nocturnal flowering plant, *J. sambac* has no genomic sequences available yet, despite its horticultural significance. We first conducted a transcriptome analysis with flower and leaf samples at three time points during flower opening (Genebank accession GHOY00000000). In the flower transcriptome, seven SWEET transporter-like, four SUC/SUT transporter-like and a number of the monosaccharide transporters-like (belong to MST family) unigenes, together with homologous genes encoding invertases and trehalose-6-phosphate synthases, are differently expressed at the time-points during the day (Figure 1). Flowers of *J. sambac* normally open right after the dusk and release fragrance at mid night, and last for approximately 26 to 30 h [40,41]. Our transcriptome roughly covers gene expressions during the major stages of flowering. These data provide an overview on important sugar utilization-related gene expression in the sink tissues (Figure 1). Specifically for the *SWEET* homologous genes, five of the seven ESTs were not found in leaves at 17:00 and 05:00, suggesting a floral-specific function (Table 1).

Using sequences of the seven *SWEET* ESTs (Table 1) as guidance, we designed primers and amplified their full-length cDNA via 5′/3′-RACE PCR. With this strategy, we successfully obtained a set of *SWEET* genes that were preferentially expressed, and might play substantial functions, during the blossom of *J. sambac*.

**Figure 2:**
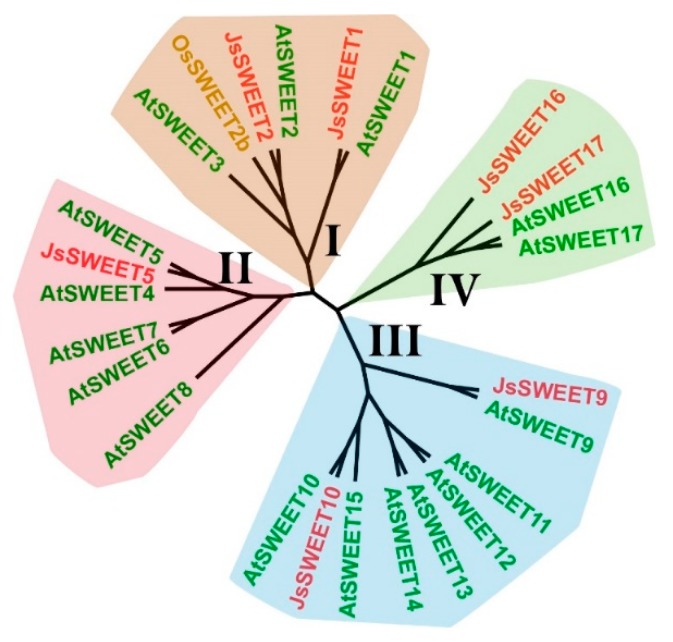
A phylogenetic tree showing the sequence relationship between the seven cloned *J. sambac* SWEET transporters (in red) and the seventeen members from Arabidopsis (in green), together with the rice homolog, OsSWEET2b (in yellow), which has a solved structure (PDB ID: 5CTH) available.

Sequence analysis revealed that these proteins represent all the four clades of the SWEET family (Figure 2 and Figure S1). Accordingly, we renamed the full-length cDNA to *JsSWEET1* (c50045_g1), *JsSWEER2* (c60775_g2), *JsSWEET5* (c41111_g1), *JsSWEET9* (c51507_g1), *JsSWEET10* (c44769_g1), *JsSWEET16* (c51046_g2), and *JsSWEET17* (c42726_g1), respectively. *JsSWEET1* shares 71.37% amino acid identity with its arabidopsis orthodox *AtSWEET1*, followed by *JsSWEET5* with 62.87% to *AtSWEET5*. The *JsSWEET2* is more closely related to *AtSWEET2* (60.43%) than to *OsSWEET2b* (53.95%) (Table S4).

### 2.2. Expression of the SWEET Genes during Flower Opening Stages

**Figure 3:**
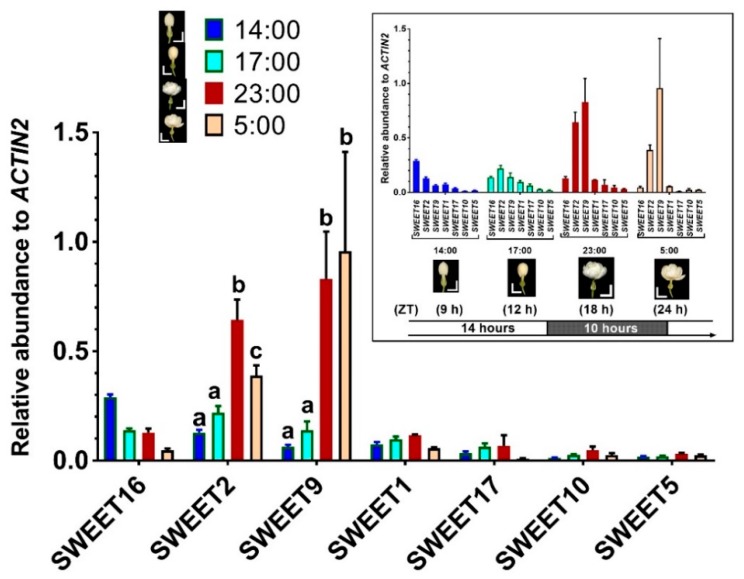
*SWEET* gene expressions during *J. sambac* flower opening. Transcript abundance relative to *JsACTIN2* was determined using quantitative RT-PCR in flower samples collected at indicated time points representing four floral stages. Significant difference between samples is indicated by different letters above the bars, which was conducted using the two-way ANOVA and multiple T-test implemented in the Prism software (version 7.04). The insert rearranges the data by the sampling time points. To correlate the time points with the circadian light/dark rhythm, the corresponding Zeitgeber time (ZT) was given below the sampling stage. Data are mean ± SD (*n* = 3 biological replicates). Scale bars = 1 cm.

The flower buds of *J. sambac* open at approximately 19:00 in the evening and remain opening during the next 26 to 30 h. The first 12 h is considered the most important period for fragrance emission (our unpublished data, [41]) and pollination [40]. To monitor the SWEET gene expression during the processes, we isolated RNA form flowers at four time points of the day at 14:00 (unopen flower buds), 17:00 (initiating loose buds), 23:00 (opening), and 5:00 (fully open) (Figure 3) and measured the relative mRNA levels using quantitative RT-PCR.

*JsSWEET10* and *JsSWEET5* were the lowest expressed genes in the *SWEET* family, while *JsSWEET2* and *JsSWEET9* had the most abundant transcript levels (Figure 3). This significantly up-regulated expression of JsSWEET9 during anthesis was similar to its orthologues in Arabidopsis [32] and Petunia [42]. Furthermore, the expression of *JsSWEET16* seemed to decrease after flower opening, reaching one-sixth at time of fully-open to that in bud stage. It was the only gene that showed a down-regulated pattern (Figure 3). Otherwise, the highly expressed transcripts of *JsSWEET9* and *JsSWEET2* were observed in fully opened flowers (Figure 3). The other four genes—namely *JsSWEET1*, *JsSWEET17*, *JsSWEET10*, and *JsSWEET5*—were expressed at relatively low level, all of which were maintained in a more or less stable fashion during the flower opening (Figure 3).

### 2.3. Subcellular Localization of the Seven SWEET Proteins

Functions of a membrane transporter depend very much on its subcellular localization. Using transient expressions of a C-terminal in-frame fusion with the green fluorescence protein (GFP), we determined the subcellular localization of the SWEET proteins in protoplasts isolated from petals of *J. sambac* flowers, as well as in arabidopsis leaf protoplasts and *Nicotiana benthamiana* epidermis cells.

**Figure 4:**
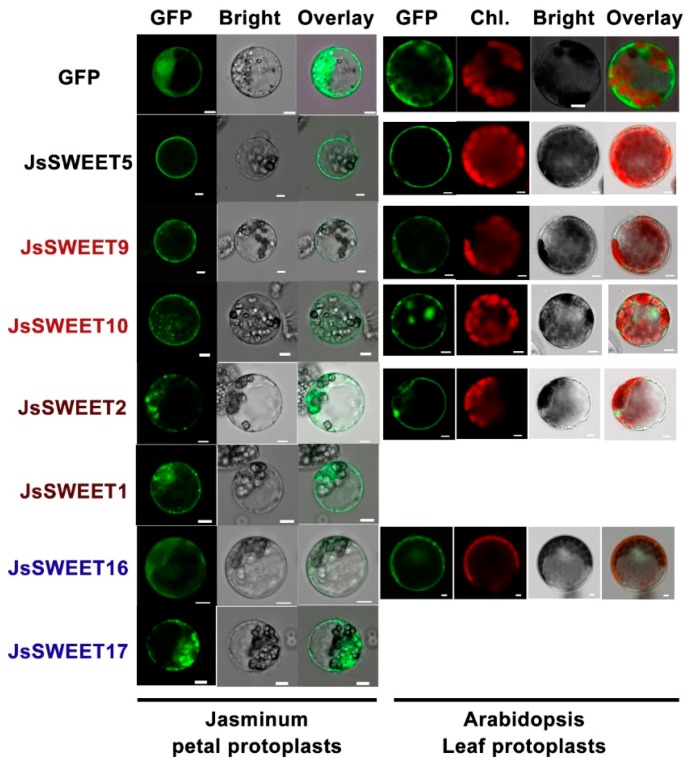
Confocal images of SWEET-GFP (green fluorescence protein) fusion proteins transiently expressed under 35S promoter in protoplasts of *J. sambac* petals and arabidopsis leaves. Images were acquired using a Leica TCS SP8 system with wavelength setting for GFP (Ex = 488 nm, Em = 507 nm) or chlorophyll (Ex = 488 nm, Em = 681 nm). Gene names are colored for clade-specific members (brown for Clade I, black for Clade II, red for Clade II and blue for Clade IV). *JsSWEET1* and *JsSWEET17* failed to express well in arabidopsis leaf protoplasts. Scale bar = 5 μm.

**Figure 5:**
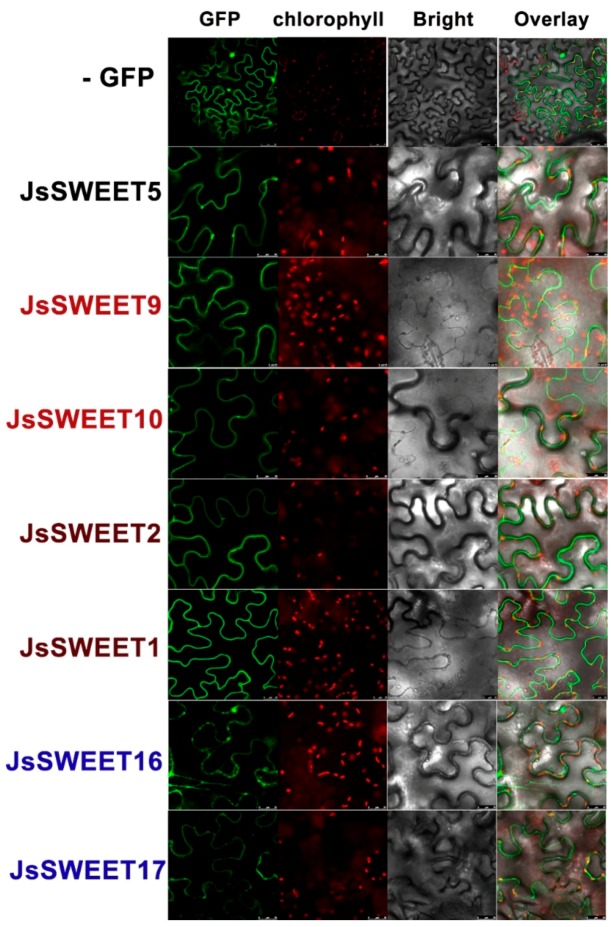
Transient expression of JsSWEET-GFP fusion proteins driven by the 35S promoter in *N. benthamiana* epidermis. Images were acquired using a Leica TCS SP8 system with wavelength setting for GFP (Ex = 488 nm, Em = 507 nm) or chlorophyll (Ex = 488 nm, Em = 681 nm). Gene names colored for clade-specific members (brown for Clade I, black for Clade II, red for Clade II, and blue for Clade IV). Scale bars, 25 (GFP control) or 50 (the rest) μm.

JsSWEET5 clearly showed a plasma membrane (PM) localization in both types of protoplasts and also in tobacco epidermis, whereas both JsSWEET9 and JsSWEET10 were localized to PM but with spot-like particles in cytosolic regions close to the PM. These spot-like particles were supposed to be Golgi or ER-PM-contact structures (Figure 4 and Figure 5). In both protoplast expressions, JsSWEET2 could be localized to the tonoplasts, but that was difficult to confirm in tobacco epidermis (Figure 4 and Figure 5). The JsSWEET1 apparently showed a PM localization but also displayed some strong intracellular signals in petal protoplasts (Figure 4), it was not expressed well in the arabidopsis leaf protoplasts. Both JsSWEET16 and 17 exhibited cytosolic or vacuolar distributions in protoplasts and in *N. benthamiana* epidermis (Figure 4 and Figure 5). JsSWEET17 was also not expressed well in arabidopsis leaf protoplasts.

Except for *JsSWEET16* and *JsSWEET17*, the transient expression of the GFP-fusions for the other five genes in *N. benthamiana* epidermis displayed apparent PM localization signals (Figure 5). These observation suggested that the subcellular localization of the SWEET proteins were sometime cell-type dependent, likely reflecting a dynamic trafficking feature for members of the family.

### 2.4. The JsSWEET5 Is a Multi-Substrates Transporter Revealed by a Yeast Complementation Assay

The yeast strain EBY4000 is defected for its endogenous 20 sugar transporter genes (17 from hexose transporter family, 1 for galactose transporter and 2 for maltose permeases), thus incapable to support cell growth on most sugar media except for maltose [43]. We expressed the cloned *JsSWEET* genes individually in this strain and examined their complementation for uptake of different sugars.

**Figure 6:**
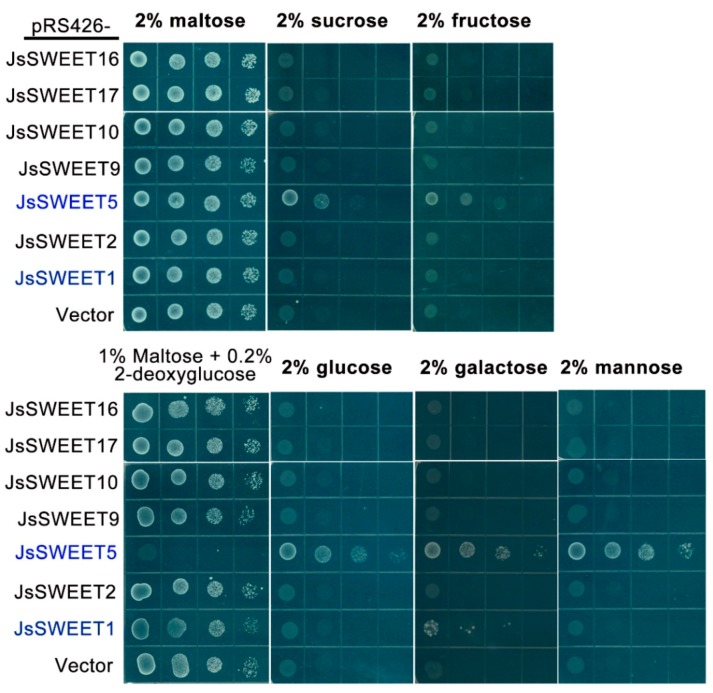
Growth complementation assay in yeast deficient strain EBY4000 by the expression of the *JsSWEETs*. Ten microliter each of a serial 10-fold diluted cell suspensions were dropped in selection media containing 0.67% Difco yeast nitrogen base with ammonium sulfate supplemented with leucine, tryptophan, histidine, and the indicated carbon sources). Maltose medium serves as a growth control for plasmid maintaining. Note that only yeast cells expressing *JsSWEET1* and *JsSWEET5* (highlighted in blue) showed growth complementation.

The yeast transformants of JsSWEET16, 17, and 2 were not able to complement the growth defect on all the sugar media tested, probably due to their vacuole or intracellular localizations (Figure 6). Similarly, expression of both JsSWEET10 and JsSWEET9 did not permit the uptake of sugars tested, indicating that either they were not properly targeted to the PM or that they might function solely as efflux transporters, as demonstrated for the arabidopsis homologue AtSWEET9 [32]. Further, in the yeast medium containing 2-deoxyglucose (2-DOG, a yeast toxic analog of glucose), yeast cells with a functional hexose importer would display a growth inhibition. Yeast cells harboring expression vectors for the above five *JsSWEETs* were not sensitive to 2-DOG (Figure 6). However, the *JsSWEET1*-expressing yeast cells showed only limited sensitivity to 2-DOG. This transformed yeast was able to grow weakly on galactose medium, meaning that JsSWEET1 was a weak galactose transporter (Figure 6).

JsSWEET5 was cable to complement the growth defect on all five sugar media–with the most on mannose and less on sucrose—and its expression rendered the yeast cells much sensitive to 2-DOG (Figure 6). Therefore, JsSWEET5 was a PM sugar transporter with a preference for hexoses over disaccharides.

## 3. Discussion

Sucrose is the predominant sugar for long-distance translocation. Being imported from the terminal phloem around the base of the flowers, sucrose is first unloaded by at least the Clade III SWEETs into the apoplasmic space, where it may be distribute to different nearby floral tissues or partially converted by cwINV to glucose and fructose. Re-uptake and transport of these sugars in the symplast among petals, androecium and gynoecium are specifically controlled by various sugar transporters. Transport activities are spacialtemporally controlled to correlate with flower development and metabolic needs. Petals are sites to attract pollinators by synthesis of colored and/or volatile secondary metabolites, by production of nectary secretion with sugar and amino acids, and importantly by generation of an osmotic-driven flower opening. All of which are sugar demanding processes [36]. To ensure successful male fertility, it has been known that specific sugar transporters are required during anthers, pollen grain development, pollen germination and pollen tube growth [44]. Furthermore, fertilization and embryo development involve intensive metabolic and signaling activities that are relevant to the functions and regulations of sugar transporters [33,36,45].

As a nocturnal pollination plant, *J. sambac* has a relatively short time-scale for floral scent production in the middle of the night [41] (our unpublished data). During this time two *SWEET* genes, *JsSWEET9* (Clade III) and *JsSWEET2* (Clade I) are up-regulated, their expression remain at very high levels throughout till dawn (Figure 3). Although not yet proved, *JsSWEET9* may be a candidate involved in nectary secretion during the flower opening, as similar to the so-far-demonstrated role of the arabidopsis orthologue *AtSWEET9* [32,42]. Orthologues of JsSWEET2 in arabidopsis and rice are known to function as tonoplast glucose transporters important for glucose sequestration in the vacuole, which is the major compartment for sugar storage [17,31]. JsSWEET2 shares 54–60% identity at the amino acids level with these two proteins and is also tonoplast localized when transiently expressed in protoplasts (Figure 4). Its expression in yeast fails to complement the uptake of glucose and other hexose or sucrose, and renders insensitive to the toxic glucose analogue 2-DOG as well (Figure 6). Thus, we speculate that JsSWEET2 may function in vacuole sugar mobilization. It will be necessary, however, to further determine its tissue or cell-type specific expression and knockout phenotype. Another Clade III member JsSWEET10 is a putative transporter in sucrose unloading.

The Clade IV members JsSWEET16 and 17 are tonoplast transporters (Figure 4 and Figure 5), their homologous in Arabidopsis play pivotal roles in controlling vacuole fructose storage [46,47,48]. *JsSWEET16* seems to be expressed more in floral bud stage whereas *JsSWEET17* maintains low but stable expression during anthesis (Figure 3). Whether they are involved in transient sugar storage or remobilization will await further studies.

The flowers of *J. sambac* also express another Clade I member, *JsSWEET1* and a single Clade II gene *JsSWEET5*, and both encode plasma membrane localized proteins. JsSWEET1 shows weak transport for galactose but rejects glucose or fructose. For a comparison, the AtSWEET1 could mediate weak transport of mannose, fructose, and galactose, in addition to its pH-dependent uptake of glucose [16]. JsSWEET5 is able to complement the yeast uptake of various sugars with stronger preference for hexose (Figure 6). The rice orthologue *OsSWEET5* is expressed in anthers [49] and the *AtSWEET5* (also called *AtVEX*, VEGETATIVE CELL EXPRESSED1) promoter-reporter translational fusions are found in mature, hydrated and germinating pollen [50]. Therefore, JsSWEET5 is very likely a sugar transporter of reproduction-relevance in *J. sambac*. Both JsSWEET1 and 5 have similar expression profiles during flowering (Figure 3), it will be interesting to further delineate their cell- or tissue-specific roles in *J.sambac*.

In summary, using floral transcriptome data as a guide, we have identified seven SWEET transporters expressed in flowers of *J. sambac*, which are well represented by the four clades of the SWEET family. Further investigations on their specific functions during reproductive development will provide helpful knowledges for crop improvement of this particular species, and is of general interests in biology of sugar transport as well.

## 4. Materials and Methods

### 4.1. Plant Materials and Growth Conditions

Three-year-old *J. sambac* var “double petals” plants were cultivated in pots (25 × 25 × 30 cm) and maintained in climate room, with 14 h photoperiod (light on at 05:00 and off at 19:00) under LED illumination (12–15 kLux) and temperature of 28/22 °C. Under our conditions, flowering usually started at c.a. 19:00 of the day, and last for the next 30 to 36 h. Flower buds were individual labeled two days priors to sampling to insure a unified developmental stage.

### 4.2. RNA-seq Library Preparation and Transcriptome Generation

For RNA isolation, leaves at 17:00 and 05:00 and flowers at 17:00, 05:00, and 11:00 were collected from three to five plants, immediately immersed into liquid nitrogen and kept in −80 °C. Samples of five leaves, or five to eight flowers, were pooled as one biological replication. Two biological replications were used for transcriptome library preparations, representing a total of 20 plants. Approximately 1 g of fresh tissue was ground into fine powders in liquid nitrogen, and the frozen powders were used for total RNA extraction with a commercial kid (Trizol extraction kit from Invitrogen). RNA degradation and contamination was monitored on 1% agarose gels and the purity was checked using the Nano Photometer^®^ spectrophotometer (IMPLEN, Westlake Village, CA, USA). The RNA integrity was assessed using the RNA Nano 6000 Assay Kit of the Agilent Bioanalyzer 2100 system (Agilent Technologies, Santa Clara, CA, USA).

A total amount of 1.5μg RNA per sample was used as input material for the RNA sample preparations. Sequencing libraries were generated using NEBNext^®^ Ultra™ RNA Library Prep Kit for Illumina^®^ (NEB, Ipswich, MA, USA) following manufacturer’s recommendations and index codes were added to attribute sequences to each sample. Briefly, mRNA was purified from total RNA using poly-T oligo-attached magnetic beads. Fragmentation was carried out using divalent cations under elevated temperature in NEB Next First Strand Synthesis Reaction Buffer (5×). First strand cDNA was synthesized using random hexamer primer and M-MuLV Reverse Transcriptase (RNase H^-^). Subsequently second strand cDNA synthesis was performed using DNA Polymerase I and RNase H. Remaining overhangs were converted into blunt ends via exonuclease/polymerase activities. After adenylation of 3′ ends of the DNA fragments, adaptor with hairpin loop structure (NEB Next Adaptor, from NEB, Ipswich, MA, USA) were added by ligation. These cDNA fragments were size-selected for 150~200 bp in length, and purified with AMPure XP system (Beckman Coulter, Beverly, MA, USA). Prior to PCR amplification, 3 μL USER Enzyme (NEB, Ipswich, MA, USA) was used to treat the size-selected, adaptor-ligated cDNA at 37 °C for 15 min followed by 5 min at 95 °C PCR. The PCR was performed with Phusion High-Fidelity DNA polymerase, Universal PCR primers and Index (X) Primer. Amplified PCR products were purified with an AMPure XP system. Finally, the quality of library was assessed on the Agilent Bioanalyzer 2100 system.

After cluster generation (using cBot Cluster Generation System of the TruSeq PE Cluster Kit v3-cBot-HS from Illumia), the library preparations were sequenced on an Illumina Hiseq platform and paired-end reads were generated. The cleaned row reads have been deposited at DDBJ/ENA/GenBank under the Accession SRR9613490 through SRR9613499. The transcriptome was assembled using Trinity [51]. Differential expression analysis was performed using the DESeq R package (1.10.1). Q value < 0.005 and log2(fold-change) > 1 was set as the threshold for significantly differential expression [52]. Gene Ontology (GO) enrichment analysis of the differentially expressed genes (DEGs) was implemented by the GOseq R packages based Walleniusnon-central hyper-geometric distribution [53] which can adjust for gene length bias in DEGs. The generated Transcriptome Shotgun Assembly project has been deposited at DDBJ/ENA/GenBank under the accession GHOY00000000. The version described in this paper is the first version, GHOY01000000.

### 4.3. Full-Length cDNA Cloning and Sequence Analysis

Full-length cDNAs of the seven *SWEET* genes were PCR cloned using the SMARTer RACE 5′/3′ Kit from TaKaRa Bio with gene-specific primers (listed in Supplemental Table S1) designed based on the transcriptome sequencing data. Complete sequences with UTR were cloned into the Gateway vector pENTYR^®^-/D-TOPO (Thermo Fisher Scientific, Shanghai, China) and subjected to Sanger sequencing. For phylogenetic analysis, sequences of the Arabidopsis 17 SWEET and the rice OsSWEET2b were download from NCBI database. Amino acid sequence alignment was conducted using MUSCLE (https://www.ebi.ac.uk/Tools/msa/muscle/) and the Neighbor-Joining tree was generated using MEGA X (https://www.megasoftware.net/) with a bootstrap number 1000.

### 4.4. Reverse Transcription Quantitative PCR (RT-PCR)

Total RNA was isolated from flower buds (at 14:00 and 17:00 of the first day) and opened flowers (at 23:00 of the first day and 05:00 of the next day) using an RNAprep pure Kit (TIANGEN Biotech, Beijing, China). First-strand cDNA synthesis was performed with the TransScript One-Step gDNA Removal and cDNA Synthesis SuperMix (TransGen, Shanghai, China). Quantitative PCR was run on a Roche LightCycler 96, using a PCR program set as an initial step of 30 s at 94 °C, followed by 40 cycles of 15 s at 94 °C, and 30 s at 60 °C. The relative expression level of target genes were calculated according to the 2^−∆∆Ct^ method as described in Schmittgen and Livak (2008) [54], with JsACTIN2 as an internal referent gene. Three independent experiments were conducted each with pooled flowers from three plants. The gene specific primers (listed in Table S3) were designed by the Primer3+ program (http://www.bioinformatics.nl/cgi-bin/primer3plus/primer3plus.cgi) (access on 5 March 2017). Each primer pair was subjected to melt curve analysis for specificity, and annealing temperature optimization was conducted by testing a range of temperatures from 55 to 70 °C.

For statistical analysis, two-way ANOVA and pair-wide multiple t-tests on means of the three biological replicates were conducted using the GraphPad Prism software (version 7).

### 4.5. Transient Expression and Subcellular Localizaton Analysis

Binary vector pK7FWG2 [55] was used for expression of the coding sequences of JsSWEETs in fusion with a C-terminal eGFP, using the Gateway cloning strategy (see Table S2 for primers used for cloning). A GFP-along expressing plasmid was also constructed for control. Expression from these plasmids was driven by the cauliflower mosaic virus 35S promoter. All the constructs were verified by sequencing.

Isolation and transformation of protoplasts from Jasminum petals and Arabidopsis leaves were carried out according to the published protocols [56,57]. For transient expression in *Nicotiana benthamiana*, *Agrobacterium* strain GV3101 harboring each expression plasmid was used to infiltrate leaves from four-week-old plants according to method in reference [58].

Fluorescence imaging was realized in a Leica SP8 Confocal Microscope, with a setting for GFP (Ex = 488 nm, Em = 507 to 530 nm) and for chlorophyll auto-fluorescence (Ex = 488 nm, Em > 670 nm). Protoplasts fluorescence were monitored at 16 h post transformation and Nicotiana leaves were observed at 2 days post infiltration.

### 4.6. Yeast Sugar Uptake Complementation Assay

The coding sequences of JsSWEETs were each subcloned into the yeast expression vector pRS426met25 with the strong promoter MET25 [59] (the cloning primers are listed in Table S2). To measure sugar uptake, we used the yeast mutant strain EBY.VW4000 (MATα *leu2-3,112 ura3-52 trp1-289 his3-Δ1 hxt1-17Δ gal2Δ stl1Δ agt1Δ mph2Δ mph3Δ*), which lacks endogenous 17 hexose transporters, 1 galactose transporter and 2 maltose permease [44]. Plasmids with or without the JsSWEET CDS were introduced into the yeast using standard PEG protocol, and transformants were selected in synthetic complete (SD) medium without uracil supplemented with 2% maltose. For uptake complementation, the maltose in the SD selection medium was replaced with respective sugar form. The yeast cultures were diluted to an OD600 of 1, and series of 10-fold dilutions were spotted on agar plates. Growth of the yeast cells was monitored after 4–6 days of incubation at 28 °C.

### 4.7. Genebank Accession

The sequence read archive (SRA) for the RNA-seq described here may be accessed with the numbers SRR9613490 through SRR9613499. Transcriptome of the *J. sambac* flowers and leaves at anthesis been deposited at DDBJ/ENA/GenBank under the accession GHOY00000000. The *JsSWEET* sequences in the Ganebank are *JsSWEET1* (MN227187), *JsSWEET2* (MN227188), *JsSWEET5* (MN227189), *JsSWEET9* (MN227190), *JsSWEET10* (MN227191), *JsSWEET16* (MN227193), and *JsSWEET17* (MN227192).

## Figures and Tables

**Table 1:** Unigenes of *SWEET* homologs preferentially expressed in flowers identified from a transcriptome analysis.

Unigene_id	Flowers @11:00 (FPKM) ^1^	Flowers @17:00 (FPKM)	Flowers @5:00 (FPKM)	Leaves @17:00 (FPKM)	Leaves @5:00 (FPKM)	Swissprot Evalue
c41111_g1	2.775	2.435	2.035	0	0	1.6 × 10^−4^
c50045_g1	15.92	14.29	11.59	0	0	1.7 × 10^−71^
c51046_g2	2.655	6.975	39.445	0	0	6.4 × 10^−86^
c51507_g1	14.165	20.89	43.425	0	0	9.6 × 10^−106^
c44769_g1	36.935	72.54	28.14	0	0.075	2.07 × 10^−66^
c42726_g1	4.95	11.43	9.86	13.32	2.62	1.2 × 10^−56^
c60775_g2	55.56	36.72	106.135	7.59	7.755	4.8 × 10^−87^

^1^ FPKM: expected number of Fragments Per Kilobase of transcript sequence per Millions base pairs sequenced.

## Supplementary Materials

The following are available online at https://www.mdpi.com/1422-0067/20/16/4001/s1, Supplemental Figure S1: Protein sequence alignment of the seven JsSWEETs and the Arabidopsis and rice homologs, Supplemental Table S1: List of Primers used for RACE cloning, Supplemental Table S2: List of Primers used for full length PCR amplification, Supplemental Table S3: List of Primers used for RT-qPCR. Supplemental Table S4: Percent Identities of JsSWEETs with homolgous from Arabidopsis and rice.
