# Supplementary material for: Cloning and Functional Assessments of Floral-Expressed SWEET Transporter Genes from Jasminum sambac
Source: Int J Mol Sci. 2019 Aug 16;20(16):4001. doi: 10.3390/ijms20164001 (PMC6719010; doi:10.3390/ijms20164001)
Supplement: Supplementary file 1 [file ijms-20-04001-s001.zip › supplementary/Supplemental Table S1-4.pdf]

Supplemental Table S1. List of Primers used for RACE cloning

Gene_ID	Primer id	Primer Sequence (5'- 3')
JsSWEET1	c50045-GSP1	GATTACGCCAAGCTTAGGACAAGAAGAAGGGCATGAACTCCA
	c50045-GSP2	GATTACGCCAAGCTTCCAATTCCCTTTTTCATTCTCAAGCGA
	c500-3-1	CCAATTCCCTTTTTCATTCTCAAGCGA
	c500-3-2	TGGCTCCAACGATTACATTCAAGAGG
	c500-5-1	AGGACAAGAAGAAGGGCATGAACTCCA
	c500-5-2	CATAAACTGATTCAATCGCTGCACCA
	C500-5race-np	TCCTCATGATGGAGAGAGGAGAAGCATACATTATG
	C500-3race-np	CTTCGGTGTTTTTGGAAATGCTACTGGATTATTTCTC
JsSWEER2	c60775-GSP1	GATTACGCCAAGCTTTGTATAATCCCAAGAATTGCTCCGACCC
	c60775-GSP2	GATTACGCCAAGCTTAGAGAATCATCAGAAGCAAATCGACCGA
	c607-5-1	GCGTCCTTCTTAAACATCCCGTACGCAA
	c607-5-2	TGCAAATACGCCAAGAACCGCAGCAA
JsSWEET5	c41111-GSP1	GATTACGCCAAGCTTTGAACAACTCCAGACAATGTCCCCAA
	c41111-GSP2	GATTACGCCAAGCTTGCTAGGACGATTGTTGGAATCTTTGGC
	c411-3-1	TCAACGGTGGAGGGTTCGCGATCGA
	c411-3-2	TCTTCCTCGTCTATGCTGGCTCGGC
JsSWEET9	c51507-GSP1	GATTACGCCAAGCTTTTTCCACGTTAAGATTTGGGTCGTCG
	c51507-GSP2	GATTACGCCAAGCTTTCAGTTGCACTCTTCAGTGCTTCATTGT
JsSWEET10	c447-3-1	CCTACTGATAATTTGTGGGTTCGGTC
	c447-3-2	CTTGCGGTGAAAGCTTCGCAGCGTGTCC
	c44769-GSP1	GATTACGCCAAGCTTTGTCAATTATTTGCTCCCGAAATTCTGG
	c44769-GSP2	GATTACGCCAAGCTTGGTTCCAATCAATTCCTTATGTGGTTGC

JsSWEET16	c51046-GSP1	GATTACGCCAAGCTTTAAAACCTGACCCATTTGCTACCTGCAC
	c51046-GSP2	GATTACGCCAAGCTTTTTCTCGCCCCAGTTGGTACTTTTTG
JsSWEET17	c42726-GSP1	GATTACGCCAAGCTTTCACGGTAAATTGCATAGAGCACCAGC
	c42726-GSP2	GATTACGCCAAGCTTTCCAGTGAAGACATTTTGGAGGATTGTG
	c427-5race-np	ACAAATCCTATTGCATTTGGTACCCCAAGAAACCAG
	c427-3race-np	TGAGAGCCTTCCTTATGTTTGTACGCTGTTG

Supplemental Table S2. List of Primers used for full length PCR amplification

Primer id	Primer Sequence (5'- 3')
Gateway cloning	
JsSWEET1-gw-5F	caccATGGGGCATGAAATCTCACATCATTAC
JsSWEET1-3r	TGGATGTGGAAGCAGGGGCTCTC
JsSWEET2-gw-5F	caccATGGTCCACACGATGTCTGAAGGCGGCTTG
JsSWEET2-3r	TGCATAGGACTCGAGTAAGGGTCTCTG
JsSWEET5-gw-5F	caccATGATGAGCAGCGACAAAGCTAGGACGATTG
JsSWEET5-3r	GGCATTAGTCCCCGGCATTTCAACC
JsSWEET9-gw-5F	caccATGGCGTTTCCAAGTGTTCAAACCTTGG
JsSWEET9-3r	CACTGCAGTCAACTGTCTATGATCATGTG
JsSWEET10-gw-5F	caccATGGCAGTTCCGTCTGGATATTGGGTTC
JsSWEET10-3r	ATTAACTTCAATTTCATTAGGCAAATTCTGATGTTTATG
JsSWEET16-gw-5F	caccATGGGGTCCCTTCTCTATTTTCCC
JsSWEET16-3r	TTGTTTGGGCTTGCTTAGGCCCA
JsSWEET17-gw-5F	caccATGATGCAATCAACCGTAAACATGATG
JsSWEET17-3r	CACTTGATTGGCTTTGTCAGCTTCATC
Yeast expression vector cloning	
pRS-JsSWEET1-5F	ATGGGGCATGAAATCTCACATCAT
pRS-JsSWEET1-3R	gagagaCTCGAGttaTTGTTTGGGCTTGCTTAGGCCCAT
pRS-JsSWEET2-5F	ATGGTCCACACGATGTCTGAAGGCGGCpRS-
JsSWEET2-3R	cccAAGCTTttaTGCATAGGACTCGAGTAAGGGTCT
pRS-JsSWEET5-5F	ATGATGAGCAGCGACAAAGCTAGGACGATTG
pRS-JsSWEET5-3R	gagagaCTCGAGttaGGCATTAGTCCCCGGCATTTCAACC
pRS-JsSWEET9-5F	ATGGCGTTTCCAAGTGTTCAAACC
pRS-JsSWEET9-3R	gagagaCTCGAGttaCACTGCAGTCAACTGTCTATGATCATGTG
pRS-JsSWEET10-5F	ATGGCAGTTCCGTCTGGATATTGGGTTC
pRS-JsSWEET10-3R	gagagaCTCGAGttaATTAACTTCAATTTCATTAGGCAAAT
pRS-JsSWEET16-5F	ATGGGGTCCCTTCTCTATTTTCCCAA
pRS-JsSWEET16-3R	gagagaCTCGAGttaTGGATGTGGAAGCAGGGGCTCT
pRS-JsSWEET17-5F	ATGATGCAATCAACCGTAAACATGATG
pRS-JsSWEET17-3R	gagagaCTCGAGttaCACTTGATTGGCTTTGTCAGCTTCATC

Supplemental Table S3. List of Primers used for RT-qPCR

Primer id	Primer Sequence (5'- 3')
JsSWEET1-qrt-600f	CGTGGAGTTCATGCCCTTCT
JsSWEET1-qrt-715r	AACCAAAACCATTGGGCACG
JsSWEET2-qrt-403f	GAACCCCCAACTCGACAACT
JsSWEET2-qrt-525r	AGGCATGAACTCAACGCTCT
JsSWEET5-qrt-198f	TCCCGATAGCCTTCTCGTCA
JsSWEET5-qrt-306r	AATCTTTTTCCGCATGGCCG
JsSWEET9-qrt-388f	GTGACGATGGTTGGATGGGT
JsSWEET9-qrt-531r	CGTGGCGTTCATAGTGAGGA
JsSWEET10-qrt-71f	ATCTTGCTCCACTGCCAACT
JsSWEET10-qrt-170r	AGCATGGCACTGAATAGAGCA
JsSWEET16-qrt-316f	TGTACTCGGAACTGCTCAGC
JsSWEET16-qrt-465r	TATGGATGTGGAAGCAGGGG
JsSWEET17-qrt-648f	GCAGTATTGGCGGGGATTCT
JsSWEET17-qrt-764r	GGCCGCTAAAGGAGAGCAAT
JsACTIN2-QRT-f	TCTCTATGGTAACATTGTCCTG
JsACTIN2-QRT-r	ATCCAGACACTGTACTTCCTCT

Supplemental Table S4. Percent Identities of JsSWEETs with homolgous from Arabidopsis and rice

	AtSWEET10	JsSWEET10	JsSWEET9	AtSWEET9	AtSWEET15	AtSWEET11	AtSWEET12	AtSWEET13	AtSWEET14	AtSWEET8	AtSWEET6	AtSWEET7	AtSWEET4
AtSWEET10	100	52.19	40.87	44.44	47.1	44.96	45.13	47.16	44.69	30.7	30.77	32.93	32.92
JsSWEET10		100	49.22	51.41	47.37	49.44	50.56	49.63	50.38	35.06	29.2	30.65	32.23
JsSWEET9			100	52.92	43.95	42.8	42.8	46.61	46.72	29.61	31.2	31.98	29.34
AtSWEET9				100	45.19	47.5	46.67	45.45	48.1	29.91	33.74	35.83	34.31
AtSWEET15					100	50	51.65	45.88	45.69	35.65	30.52	32.93	35.54
AtSWEET11						100	84.1	57.04	55.4	35.53	29.15	30.89	32.64
AtSWEET12							100	57.5	57.25	35.96	32.65	33.2	34.18
AtSWEET13								100	75.27	33.92	30.52	31.85	29.46
AtSWEET14									100	34.65	29.75	32.37	31.91
AtSWEET8										100	38.4	42.13	45.11
AtSWEET6											100	73.64	46.61
AtSWEET7												100	46.77
AtSWEET4													100
JsSWEET5													
AtSWEET5													
JsSWEET16													
JsSWEET17													
AtSWEET16													
AtSWEET17													
AtSWEET3													
JsSWEET1													
AtSWEET1													
OsSWEET2b													
JsSWEET2													
AtSWEET2													

	JsSWEET5	AtSWEET5	JsSWEET16	JsSWEET17	AtSWEET16	AtSWEET17	AtSWEET3	JsSWEET1	AtSWEET1	OsSWEET2b	JsSWEET2	AtSWEET2	
AtSWEET10	31.14	31.17	33.19	34.8	33.78	35.37	33.61	34.07	35.5	31.42	31.86	31.28	
JsSWEET10	33.04	31.33	32.31	34.65	30.77	33.62	34.82	35.37	37.18	31.25	30.67	30.09	
JsSWEET9	34.48	28.94	32.63	33.05	34.21	33.47	32.27	34.32	35.02	31.56	32.6	33.33	
AtSWEET9	32.62	30.08	34.32	36.86	32.31	35.83	32.65	32.63	32.34	34.96	33.04	31.14	
AtSWEET15	31.88	34.48	37.39	36.68	35.45	35.96	33.33	32.17	35.62	33.63	36.68	30	
AtSWEET11	33.77	32.03	37.55	35.81	36.2	36.96	30.04	34.5	37.93	33.93	35.68	32.89	
AtSWEET12	32.89	33.33	36.24	35.81	34.84	35.65	31.54	35.37	36.96	35.27	36.12	35.09	
AtSWEET13	31.28	30.43	38.94	38.05	34.39	34.93	31.17	37.17	40.77	36.32	34.82	34.22	
AtSWEET14	32.89	32.75	37.89	36.12	33.33	34.35	31.95	37	39.57	35.27	34.22	34.07	
AtSWEET8	44.68	44.26	34.91	39.13	38.64	37.83	34.91	35.9	40.52	29.69	31.88	31.74	
AtSWEET6	46.41	45	32.19	36.36	34.68	34.91	34.92	41.7	40.91	34.93	35.65	34.63	
AtSWEET7	48.72	48.95	32.9	38.43	39.73	37.12	35.34	42.92	43.33	35.37	35.65	33.77	
AtSWEET4	54.43	58.33	36.68	37.89	39.82	38.7	33.61	41.56	41.45	35.68	36.56	32.02	
JsSWEET5	100	62.87	37.55	38.33	37.27	38.86	34.2	37.23	41.92	33.92	32.6	31.14	
AtSWEET5		100	34.93	36.56	37.1	38.26	32.91	37.23	40.43	32.6	33.48	30.7	
JsSWEET16			100	52.3	47.09	49.36	36.36	38.91	38.96	35.71	34.93	31.3	
JsSWEET17				100	54.46	58.37	34.2	36.4	41.05	35.71	32.91	30.74	
AtSWEET16					100	72.17	35.81	39.46	37.84	34.26	33.49	31.48	
AtSWEET17						100	34.87	39.22	39.66	37.1	36.04	29.6	
AtSWEET3							100	42.74	41.63	40.18	38.67	38.05	
JsSWEET1								100	71.37	39.65	43.04	39.32	
AtSWEET1									100	44.44	46.46	41.85	
OsSWEET2b										100	53.95	53.71	
JsSWEET2											100	60.43	
AtSWEET2												100	
