# Supplementary figures and images for: Cloning and Functional Assessments of Floral-Expressed SWEET Transporter Genes from Jasminum sambac
Source: Int J Mol Sci. 2019 Aug 16;20(16):4001. doi: 10.3390/ijms20164001 (PMC6719010; doi:10.3390/ijms20164001)

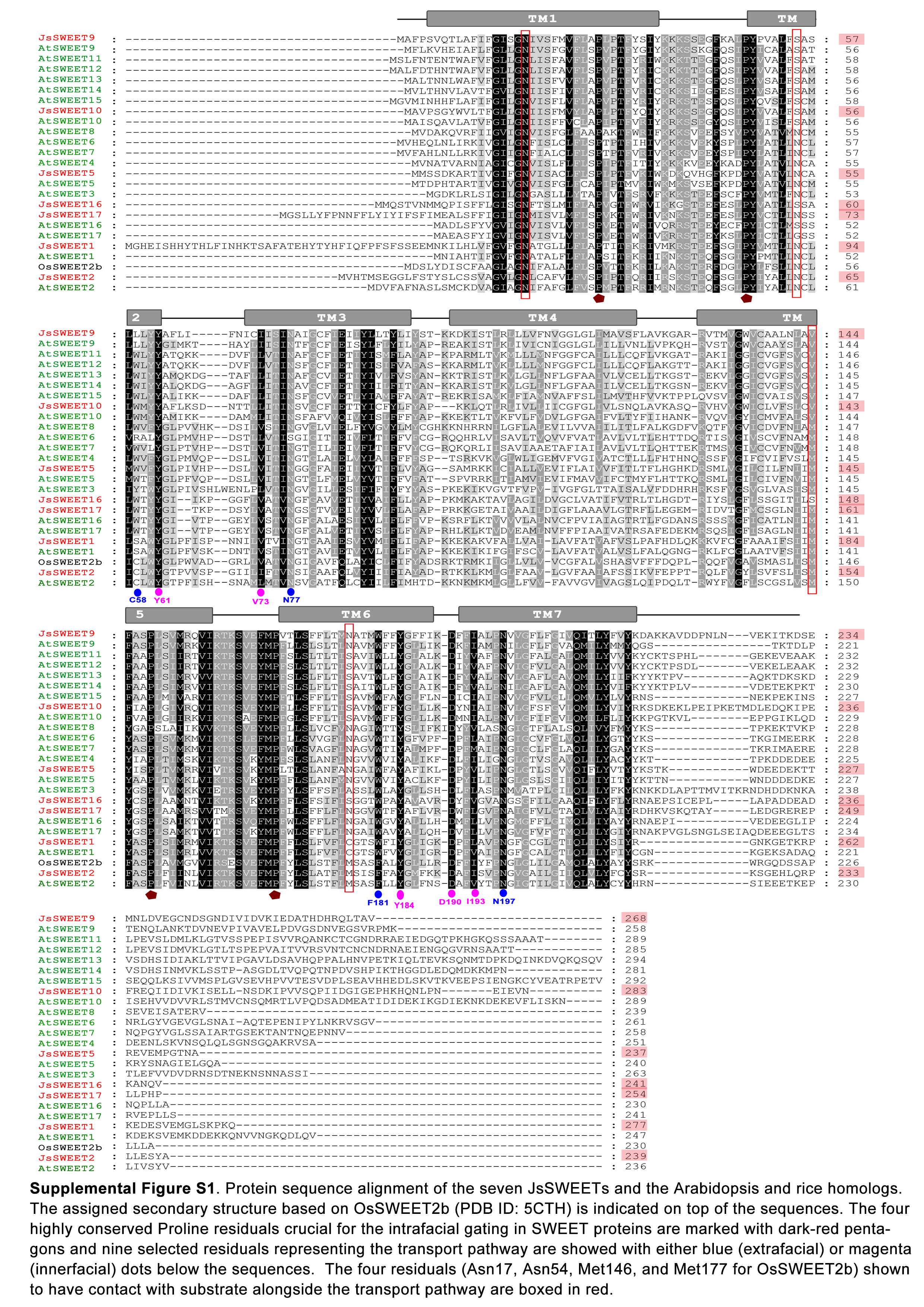

Supplement: Supplementary file 1 [file ijms-20-04001-s001.zip › supplementary/Supplemental Figure S1.jpg]
